# Bupropion and Citalopram in the APP23 Mouse Model of Alzheimer's Disease: A Study in a Dry-Land Maze

**DOI:** 10.1155/2012/673584

**Published:** 2012-09-29

**Authors:** Katharina L. Neumeister, Matthias W. Riepe

**Affiliations:** ^1^Division of Mental Health & Old Age Psychiatry, Psychiatry II, Ulm University, 89312 Günzburg, Germany; ^2^Department of Geriatrics & Old Age Psychiatry, Psychiatry II, Ulm University, Ludwig-Heilmeyer-Straße 2, 89312 Günzburg, Germany

## Abstract

*Background*. Incipient Alzheimer's disease is often disguised as depressive disorder. Over the course of AD, depressive symptoms are even more frequent. Hence, treatment with antidepressants is common in AD. It was the goal of the present study to assess whether two common antidepressants with different mechanisms of action affect spatial learning in a transgenic animal model of Alzheimer's disease. *Methods*. We assessed spatial memory of male wild-type and B6C3-Tg(APPswe,PSEN1dE9)85Dbo (APP23) transgenic animals in a complex dry-land maze. Animals were treated with citalopram (10 mg/kg) and bupropion (20 mg/kg). *Results*. Moving and resting time until finding the goal zone decreased in 4.5-month-old sham-treated wild-type animals and, to a lesser extent, in APP23 animals. Compared with sham-treated APP23 animals, treatment with bupropion reduced resting time and increased speed. On treatment with citalopram, moving and resting time were unchanged but speed decreased. Length of the path to the goal zone did not change on either bupropion or citalopram. *Conclusion*. Bupropion increases psychomotor activity in APP23 transgenic animals, while citalopram slightly reduces psychomotor activity. Spatial learning per se is unaffected by treatment with either bupropion or citalopram.

## 1. Introduction

Alzheimer's disease (AD) is a progressive neurodegenerative disease characterized by accruing cognitive and noncognitive deficits. Among the latter, aberrant motor behavior with either overall slowing or hyperactivity, and depression are common symptoms [1–5]. 

In patients with AD, presence of apathy as indicated by decreased motor activity and decreased agitation/hyperactivity as indicated by increased motor activity is associated with faster functional and cognitive decline [5–7]. Levels of dopamine, noradrenaline, and serotonin are decreased in patients with AD [8–11]. In animal models of AD, dopamine, noradrenaline, and serotonin all have been reported to improve cognitive functioning [12, 13]. 

Bupropion is a monoamine reuptake inhibitor selective for dopaminergic and to a lesser extent noradrenergic neurotransmission but has no effect on serotonergic neurotransmission [14]. Bupropion improved visual memory in patients with major depressive disorder [15]. In rodent animal models, bupropion significantly decreased the duration of immobility on the forced swim test [16] and increased locomotor activity in freely moving animals [17].

Citalopram is a potent selective inhibitor of serotonin reuptake [18] and increases extracellular serotonin concentrations in the hippocampus of rats [19] but has no effect on the uptake of noradrenaline and dopamine [20]. Citalopram was recently reported to improve spatial memory in rats [21] and to decrease immobility time in the forced swim test [22].

A standard paradigm for the assessment of cognition in animal research is the investigation of spatial learning in water and dry-land mazes [23–25]. While early work on spatial orientation in rodents was performed in complex dry-land mazes [24], since the mid 1980s complex spatial tasks predominantly are assessed in water mazes [25]. However, using water mazes poses an additional stress on animals [26]. Hence, water maze learning likely not only assesses cognitive processes of spatial memory but is confounded by noncognitive components such as anxiety. It was the goal of the present study to assess whether common substances used for treatment of depression in humans affect spatial learning in a transgenic animal model of AD.

## 2. Methods

Experiments were performed under the animal protocol number 1006 (Regierungspräsidium Tübingen).

### 2.1. Animals and Treatment

Three groups of 4.5-month-old male APP23 mice (Charles River) and one group of same aged male wild-type mice were used in this study. Three or four animals were housed in a cage and were maintained on a 12 h light/dark cycle in a temperature (22 ± 2°C) and humidity (55 ± 5%) controlled room similar to previous protocols [23, 27, 28]. 

One group of APP23 mice was treated with a daily i.p. injection of bupropion (20 mg/kg body weight), another with citalopram (10 mg/kg body weight), respectively, starting fourteen days prior to onset of experiments. One group of APP23 mice and wild-type mice were NaCl-sham treated with the same protocol.

### 2.2. Maze and Behavioural Testing

**Figure 1:**
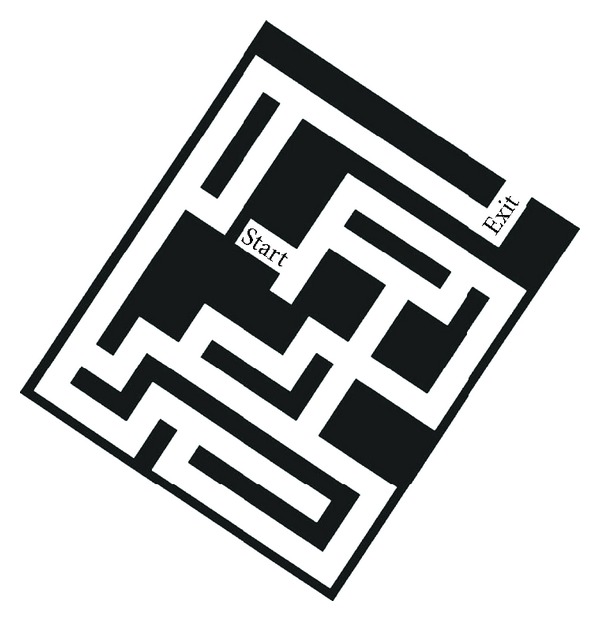
Aerial view of the complex maze. Animals were placed in the start zone. A video-tracking system (cf.  Section 2) registered the location of the animals' position at a frequency of 1 Hz.

To assess spatial memory, we used a complex maze that has been described previously [23] (Figure 1). Starting on the date of the last treatment, animals were trained four times a day for three consecutive days and two times on the fourth day. They had a maximum time of 300 s to find the exit, where they were rewarded with a food pellet. The behavioural testing took place between 11:00 AM and 5:00 PM. Different parameters, for example, duration, moving and resting time were recorded by a tracking system (Multitrack, Accuscan, USA).

### 2.3. Statistical Analysis

All statistical analyses were carried out using the statistics program SPSS (SPSS 17.0 for Windows, SPSS Inc. IL, Chicago, 60606). Statistical significance was accepted at *P* < 0.05.

## 3. Results

### 3.1. Sham-Treated Groups

**Figure 2:**
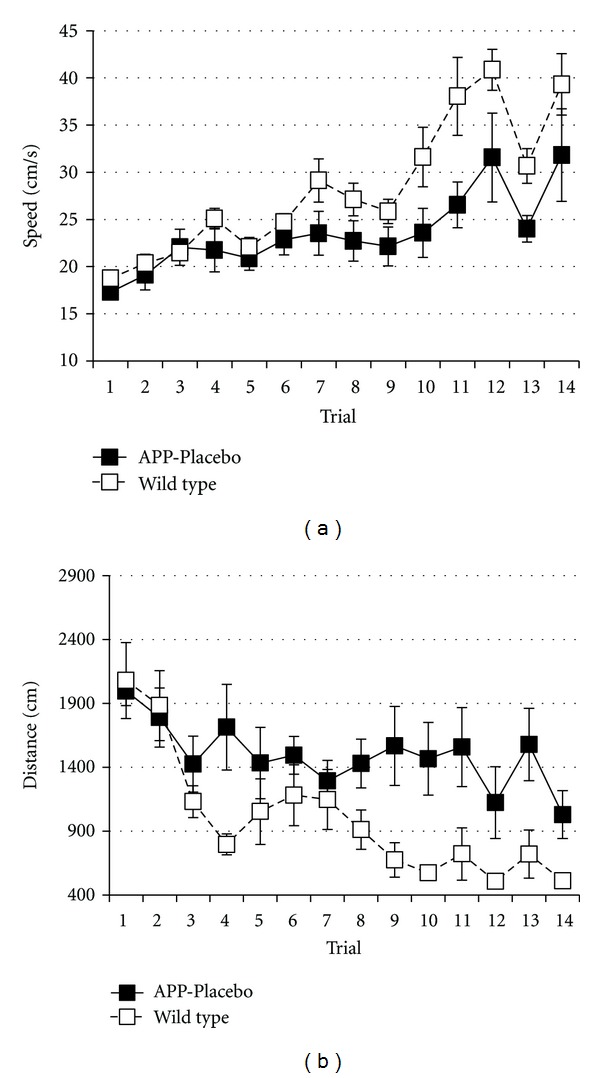
Running speed (a) and learning curve for the distance (b) to escape from the complex maze. Values represent means and standard errors for a group of wild-type animals (*n* = 7, open squares) and APP23 animals (*n* = 6, filled squares), both sham treated. Values represent means ± standard errors.

Both wild-type and APP23 animals improved their performance during repeated exposure to the dry-land maze as shown by a significant effect of trial on total time (two-way ANOVA; *F*_13,154_ = 6.751, *P* < 0.001), resting time (two-way ANOVA; *F*_13,154_ = 5.042, *P* < 0.001), and moving time (two-way ANOVA; *F*_13,154_ = 9.157, *P* < 0.001). Both groups increased their running speed over all trials (two-way ANOVA; *F*_13,154_ = 11.577, *P* < 0.001) with wild-type animals more so than APP23 animals (two-way ANOVA; *F*_1,154_ = 28.676, *P* < 0.001) (Figure 2(a)). To catch the efficacy of spatial learning, it is thus necessary to determine the path length for reaching the goal zone. Both wild-type and APP23 animals improved their performance during repeated exposure to the dry-land maze as shown by a significant effect of trial on distance (two-way ANOVA; *F*_13,154_ = 4.942, *P* < 0.001) (Figure 2(b)). Path length was shorter for wild-type animals than for APP23 animals (two-way ANOVA; *F*_1,154_ = 37,767, *P* < 0.001). 

### 3.2. Antidepressant-Treated Groups

**Figure 3:**
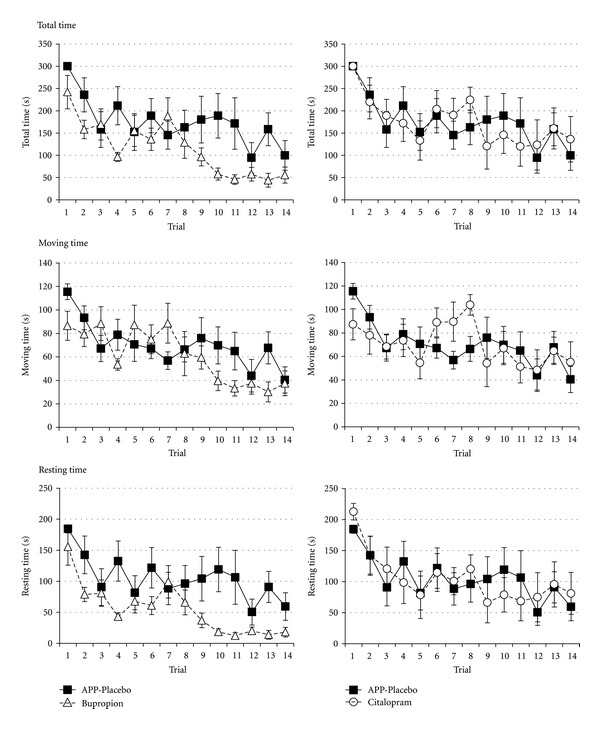
Learning curve for the time to escape from the complex maze in APP23 animals sham treated (*n* = 6, filled squares), APP23 animals treated with bupropion (*n* = 7, filled triangles), or APP23 animals treated with citalopram (*n* = 6, filled circles). Values represent means ± standard errors.

To assess the effect of treating APP23 animals, we performed a two-way ANOVA with treatment groups sham treatment, bupropion treatment, and citalopram-treatment. There was a significant effect of treatment group on total time (*F*_2,224_ = 12.794, *P* < 0.001) with Fisher LSD multiple comparison testing indicating significant differences (Table 1, Figure 3). Likewise, a significant effect of treatment group was found on resting time (*F*_2,224_ = 18.103, *P* < 0.001) with Fisher LSD multiple comparison testing indicating significant differences for treatment with bupropion but not for citalopram treatment (Table 1, Figure 3). Contrary, no overall effect of treatment was found on moving time (*F*_2,224_ = 2.224, *P* = 0.111; Table 1, Figure 3). 

**Figure 4:**
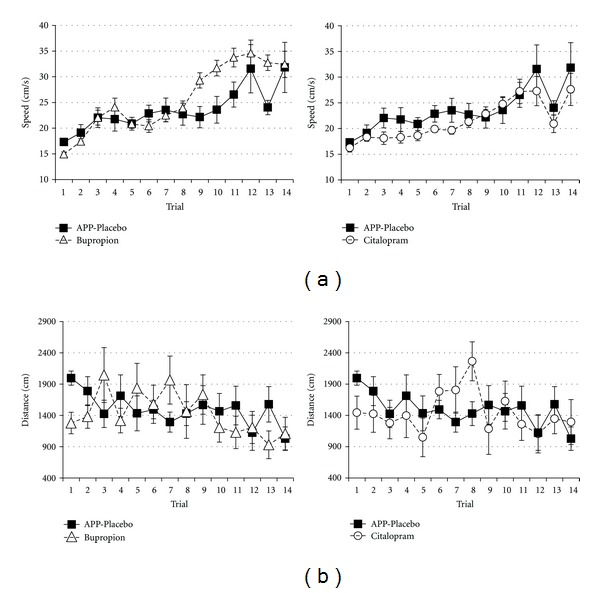
Running speed (a) and learning curve for distance (b) to escape from the complex maze in APP23 animals sham treated (*n* = 6, filled squares), APP23 animals treated with bupropion (*n* = 7, filled triangles), or APP23 animals treated with citalopram (*n* = 6, filled circles). Values represent means ± standard errors.

Analysis of running speed featured significant treatment differences (*F*_2,224_ = 17.507, *P* < 0.001) with Fisher LSD multiple comparison testing showing that bupropion-treated animals run faster while citalopram-treated animals run with less speed than sham-treated animals (Table 2, Figure 4(a)). Further there was no significant effect of treatment group on distance (*F*_2,224_ = 0.124, *P* = 0.883) (Table 2, Figure 4(b)).

## 4. Discussion

Maze studies are an established means to investigate spatial learning in experimental animals [24], both in water mazes [25] and dry-land mazes [23]. Compared to wild-type animals, the total, moving, and resting time to find the goal zone were decreased in APP23 transgenic animals and the running speed was increased. However, path length was also shorter in wild-type than APP23 animals. We conclude that the difference between wild-type and APP23 animals reflects both, better spatial learning and higher psychomotor activity. This is in good harmony with previous studies showing diminished learning in middle-aged APP23 animals [29, 30]. Reduced running speed in APP23 is in good harmony with a previous study with reduced psychomotor activity in another transgenic mouse model of AD, the APP/PS1 transgenic model [31]. 

Citalopram has been used in dosages from 0.01 mg/kg to 8 mg/kg. The effects on spatial cognition remain ambiguous and depend on dosage, paradigm, species, and the interaction thereof. At low dosages, citalopram did not affect spatial learning or even improved it [21] but had negative effects in dosages higher than 4 mg/kg in rats [32]. At moderate dosages of about 5 mg/kg, citalopram decreased immobility time in the forced swim test [22]. To our knowledge, the present study is the first to address the effects of citalopram on spatial learning in a transgenic mouse model of AD. In this model, high dosages of citalopram did not change path length, total, moving, or resting time, while running speed was slightly decreased. We interpret this such as to indicate that spatial learning per se is unaffected but that psychomotor activity is slightly decreased. This is in good harmony with previous reports showing either a slight decrease of psychomotor activity on administration of comparable dosages of citalopram [33] or unchanged locomotor activity [34]. At least in clinical studies, no benefit on cognition was found on treating AD patients with citalopram [35, 36].

On treatment of APP23 animals with bupropion, resting time decreased and running speed increased while length of path and moving time remained unchanged. We interpret this finding such as to indicate that bupropion does not improve spatial learning per se but that it increases psychomotor activity. This is in good harmony with a previous study in mice showing an increased locomotor activity in dosages from 1 mg/kg to 20 mg/kg [37].

 Altogether, bupropion and citalopram differentially affect psychomotor activity in the APP23 transgenic mouse model of AD while spatial learning per se is unaffected. 

## Figures and Tables

**Table 1:** Two-way ANOVA for total, resting and moving time for APP23 animals with treatment groups sham treatment (sham), bupropion treatment (bup), and citalopram treatment (cit). Post hoc multiple comparison testing (Fisher LSD) with *P* values for comparison of differences between groups. *Indicates statistical significance.

	Mean	SEM		Between group *P* value	
				To sham	To bup
Total time					
sham	174.8	11.3			
bup	116.5	8.8		<0.001*	—
cit	174.2	11.6		0.964	<0.001*
Resting time					
sham	104.9	8.1			
bup	55.1	5.6		<0.001*	—
cit	103.8	8.7		0.912	<0.001*
Moving time					
sham	69.9	3.7			
bup	61.4	3.7		0.081	—
cit	70.4	4.0		0.926	0.066

**Table 2:** Two-way ANOVA for running speed and distance for APP23 animals with treatment groups sham treatment (sham), bupropion treatment (bup), and citalopram treatment (cit). Post hoc multiple comparison testing (Fisher LSD) with *P* values for comparison of differences between groups. *Indicates statistical significance.

	Mean	SEM		Between group *P* value	
				To sham	To bup
Running speed					
sham	23.6	0.8			
bup	25.8	0.7		0.003*	—
cit	21.5	0.6		0.006*	<0.001*
Distance					
sham	1492.2	66.2			
bup	1441.5	82.3		0.642	—
cit	1447.4	84.0		0.693	0.957
